# Tribological Behavior of Nanocomposites Based on UHMWPE Aged in Simulated Synovial Fluid

**DOI:** 10.3390/polym10111291

**Published:** 2018-11-21

**Authors:** Annamaria Visco, Samy Yousef, Cristina Scolaro, Claudia Espro, Mariateresa Cristani

**Affiliations:** 1 Department of Engineering, University of Messina, C.da Di Dio, 98166 Messina, Italy; cscolaro@unime.it (C.S.); espro@unime.it (C.E.); 2 Institute for Chemical-Physical Processes CNR-IPCF, Viale Ferdinando Stagno d’Alcontres, 37, 98158 Messina, Italy; 3 Department of Production Engineering and Printing Technology, Akhbar Elyom Academy 6th of October, Giza 12511, Egypt; ahmed.saed@ktu.lt; 4 Department of Production Engineering, Faculty of Mechanical Engineering and Design, Kaunas University of Technology, 51424 Kaunas, Lithuania; 5 Department of Chemical, Biological, Pharmaceutical and Environmental Sciences, V. Annunziata, 98168 Messina, Italy; mcristani@unime.it

**Keywords:** UHMWPE, nanocomposites, ageing, tribological behavior

## Abstract

Ultra High molecular weight polyethylene (UHMWPE) suffers wear degradation in total joint replacements and it needs to be improved. Thus, we enhanced wear resistance of UHMWPE with carbon nanofiller and paraffin oil and studied its tribological behavior in Simulated Synovial Fluid (SSF) for 60 days at 37 °C to reproduce the conditions of a real joint. Ageing in biological fluid accelerates the wear action but nanocomposite exhibited a higher wear resistance compared to UHMWPE because of its higher structural homogeneity. Carbon nanofiller closes the porosity of UHMWPE hindering SSF to penetrate inside. Wear resistance of the nanocomposite with 1.0 wt.% of CNF improved of 65% (before ageing) and of 70% (after 60 days in SSF) with respect to pure UHMWPE.

## 1. Introduction

The Ultra High Molecular Weight Polyethylene (UHMWPE) has been considered the standard material for Joint Replacement (JR) in artificial knees and hips prosthesis for more than half a century for its high strength, chemical resistance, and bio-compatibility [1,2]. Artificial replacements in the human body are supposed to last for 15–25 years in most cases. However, if a failure occurs by wear debris generation, the implant life time can be shorter for infection or instability and it needs revision surgery [3,4,5]. Therefore, many wear tests are necessary to assess the wear rate of JR and to estimate their service time. These studies have supposed that mechanical properties of UHMWPE are homogeneous throughout the component and unchanged with time of implantation [6,7,8,9]. Anyway, it must be taken into account that UHMWPE undergoes deterioration in a physiological environment due to chemical reactions that accelerate the wear mechanisms [10].

Normally, the in vivo work of a joint is conducted in a Synovial Fluid (SF) environment that is the natural lubricant, which reduces wear rate in joints for its high viscosity (like mineral oil) and consists of many biological molecules (proteins, lipids, and polysaccharides which form lubricating films) [11,12,13,14]. Proteins of SF can be absorbed by UHMWPE enhancing the lubrication by means of the presence of a hydrophilic low-shear–strength fluid layer upon the polymer [15]. However, it is difficult to use SF in laboratory experiments because extracting it from the human body is not permitted by law in many countries and it is not available in large quantities [16]. Calf or Bovine Serum (BS) is recommended by American Standard Testing Materials (ASTM) as the most appropriate alternative for the in vitro tests, due to its properties which are very similar to the human one [17]. Anyway, there is not full agreement concerning this choice because proteins concentration can be very different having a dramatic effect on friction and wear of the tribological pairs used in the prosthetic joints. Proteins are primary constituents of synovial fluid in human joints with other organic components, such as Hyaluronic Acid (HA) and lubricin [18]. HA is one of the major components of articular cartilage, together with albumin; it is used as a coating to provide good tribological properties because it is responsible for the high viscosity [17,19,20]. In order to reproduce the degradation effects of synovial fluid, Shirong Ge et al. (2011) [10] studied the effect of the biodegradation of UHMWPE in Simulated Body Fluid (SBF) on the mechanical properties and tribological behavior for up to one year. These authors observed as biodegradation of UHMWPE results from the interaction of oxidative degradation, hydrolysis, biodegradation and tribofatigue and they highlighted that few papers are present in literature about this issue. Azam et al. [21] highlighted the importance of accelerate ageing studies for estimating the service life of UHMWPE. They carried out an FTIR comparative study on commercially available UHMWPE (i.e., GUR 1020, GUR 1050 and laboratory grade UHMWPE from Sigma Aldrich) aged at 80 °C in air for three weeks and highlighted that GUR 1020 suffer more the oxidative degradation than the other two materials. Anyway longer ageing studies are necessary to completely evaluate this aspect.

Generally, carbon nanofiller can be employed as lubricant to increase the wear resistance of component in friction units [22]. UHMWPE reinforced with carbon nanofibers (CFR-UHMWPE, named Poly II) was used in orthopedic implants in the 1970s for THA/TKA (Total Hip or Knee Arthoplasty) [23]. However, this composite was discontinued due to evidence on reduced crack resistance, rupture of the fibers on the surface, and other issues [4]. The improvement in the incorporation methods developed in the more recent years, the intrinsic properties and cytocompatibility of the carbon nanofiller, let to a reconsideration of these nanocomposites [22,24]. Experimental research studies carried out over the period between 1999 and 2017, showed a reduction in wear rate within the range of 54–58% of nanocomposites reinforced with 0.5 wt.%–5 wt.% of carbon nanofiber [4,25]. In order to avoid filler agglomeration during the mixing process and to enhance even more the lubricant action of carbon nanofiller, we applied a paraffin oil assisted mixing method to produce UHMWPE based nanocomposites. With this aim, in our previous paper we have established the optimal preparation technique and optimal composition (in terms of Carbon Nano Filler-CNF amount) of a nanocomposite based on the pure biomedical grade UHMWPE-GUR 1020 [26]. Paraffin Oil (PO) is added in the nanocomposite to decrease the viscosity of the polymer and improve the CNF dispersion; besides, it reduces the fusion defects during its processing, lowering the grain boundaries amount in polyethylene [26,27]. Galetz and Wood have tested the wear behavior of similar nanocomposites, prepared by PO assisted mixing techniques [13,27]. Comparing the wear behavior of our sample with those obtained by these two Authors, we had wear comparable and/or higher features (especially in terms of yielding and ductility). Additionally, we do not have any macromolecular break and consequent molecular weight decreasing during the melt mixing with ball milling, as checked by Galetz et al. [13]. The process was fast since we do not need to remove the PO avoiding long and expensive chemical processes.

The innovative aspect of the use of this nanocomposite material in tribological applications has been also discussed: CNFs have shown an effect comparable to that obtained with graphene and are very attractive for their low cost [28,29]. In fact, graphene lubricant exhibits a reduction in wear rate of 1.5–4.5 times, depending on filler load (0.1 wt.%–1.0 wt.%) while our nanocomposites had a maximum reduction was of 1.7 times (in air media) and of 5.8 times (in lubricant media) with respect to pure polyethylene suggesting that the experimental result obtained with carbon nanofiller is comparable to that obtained with the graphene [4].

In the present research, we completed the study of the possible application of these nanocomposites with the analysis of the tribological behavior of nanocomposites (based on UHMWPE with 2 wt.% of PO and with 0.5 or 1.0 wt.% of CNF) during the ageing in Simulated Synovial Fluid (SSF) for 60 days at 37 °C, in comparison with pure UHMWPE. It has been evaluated by means of wear tests, with morphological, mechanical and calorimetric characterization analyses performed before and during the ageing period in SSF.

## 2. Materials and Methods

Materials Medical grade GUR1020-UHMWPE powder (code “UH”) was the reference sample (average molecular weight of 2–4 × 10^6^ g/mol, *d* = 0.93 g/cm^3^ without calcium stearate, supplied by Ticona, Sulzbach, Germany). Nanocomposites were obtained by mixing pure UH with 2 weight % of pharmaceutical grade Paraffin Oil (Sella Pharmaceutical and Chemical Laboratory, Schio (Vi), Italy) with 0.5 weight% and 1.0 weight% of Carbon Nano Filler (code: “UH-0.5CNF” and “UH-1.0CNF”, respectively). These amounts have been chosen according to a previous investigation [21]. CNF powder (Zoltek, Bridgeton, Missouri-USA) was obtained by milling short carbon fibers in a ball milling (mod.-MM301, Retsch, Haan, Germany) 30 cycles of 10 min at 50 rpm. More details of the reference sample and nanocomposites preparation were already described in a previous paper [25]. In particular, polymeric sheets (60 mm × 60 mm, and 1 mm and 2 mm thick) were prepared by compression moulding in a laboratory press at 200 °C for 20 min at 20 MPa pressure in copper die between two Teflon^®^ sheets, 0.1 mm thick. All the samples (pure UH and the two nanocomposites UH-0.5CNF and UH-1.0CNF) have been subjected to a biological ageing to study the change of its mechanical performance during a period of 60 days at 37 °C, in Simulated Synovial Fluid (SSF) that was formed by dissolving 0.3 wt.% HA in phosphate buffered saline solution (pH 7.4) as reported in Table 1 [30].

All the samples were sterilized by means of 0.22 µm polycarbonate filters and ethyl alcohol. Each sample was added of 15 mL SSF and kept for 7, 14, 28 and 60 days at 37 °C. The sterility maintenance was evaluated by visually observing the absence of turbidity.

Specific wear rate measurements: a pin-on-disc wear tester (Prototype, Messina, Italy) was used to perform wear resistance measurements, at room temperature, with Simulated Synovial Fluid (SSF) as lubricant in order to approximate the chemical environment of a human joint. The samples had square shapes (20 mm × 20 mm and 2 mm thick). In order to obtain a significant weight loss and improve the final result accuracy, it was performed an “accelerate wear test” with a very rough pin made by ruby corundum grinding stones (M.2145), 3 mm in diameter. The pin on disc system and its components are detailed in a previous paper [21]. The optimum test conditions, constrained by a noticeable weight loss and reliable for specific wear rate calculation, were: testing load 30 N and 60 rpm, during a test time of 120 min. The chosen wear trajectory was of circular shape to grant uniform stress upon the samples and to produce reliable and repeatable wear tracks, in terms of length and thickness. The specific wear rate *W_s_* (mm^3^/Nm) of pure UH and of nanocomposites was calculated by the following equations [31]:

(1) $$W_{s}=\frac{\Delta m}{P\cdot F_{n}\cdot L}$$

(2) L=πd

where *Δm* (mg) is the mass loss of the specimen and measured using a high sensitivity electronic weighing balance (accuracy: 10^−4^ g), P (g/mL) is the density (see Table 2), *F*_n_ (N) is the normal load and *L* (m) is the total sliding distance. *L* that can be calculated by Equation (2), where *d* (mm) is average diameter of each wear track. Three tracks of different diameter were obtained after the two hours both on the front face of the sample (a small and a large size wear track) and on the other side (a medium size track). *W_s_* value for each sample was the average of the three wear tracks.

Density value of each sample has been calculated by means of a Balance (mod. Explorer pro EP 214C, precision of 0.1/1 mg, ASTM D1505 international standard, Ohaus Corporation, Pine group, NJ-USA) equipped as a hydrostatic balance that follows the Archimede’s principle. The density (ρ) is evaluated from dry (*P*_dry_) and wet (*P*_wet_) weight measurements of the sample before and after the immersion in ethanol at room temperature for five minutes (whose density, ρ_eth_, is 0.790 g/cm^3^), as indicated in the following equation:

(3) $$\rho =\frac{P_{\mathrm{dry}}}{P_{\mathrm{dry}}-P_{\mathrm{wet}}}\rho_{\mathrm{eth}}$$

The final density value of each sample was the average of *n* = 3 measurements.

Mechanical tensile test: was performed by using a Lloyd Universal Testing Machine, model LR10K (Ametek-Lloyd Instruments Ltd, Fareham Hampshire, UK), with a crosshead speed of 10 mm/min and a load cell of 500 N. The sample’s geometry used for tensile stresses was made according to the ASTM 638 M-3 international standard (60 mm total length, 10 mm useful length, 2.5 mm minimal width, 1 mm thickness) by using a manual Ray-Ran cutter. The medium value of each parameter resulted from the average of six measurements performed for each test. The parameters investigated in particular were: tensile modulus, yield strength and strain, stress and strain at break.

A Scanning Electron Microscope Zeiss Crossbeam 540 FEG-SEM (Carl Zeiss Microscopy GmbH, Jena, Germany) was used to carry out morphological investigations of the UH and of the UH-1.0CNF nanocomposite surface. For the SEM investigations, samples were coated in vacuum with a very thin Chromium film to make them electrically conductive. The samples were cut and mounted on an aluminum stab with a conductive adhesive film. The electron acceleration voltage was of 10 kV.

Differential Calorimetric Analysis (DSC, TA Instruments, New Castle, DE) was performed by heating each sample and its reference from 30 °C to 230 °C with a rate of 10 °C/min. Three measurements for each sample gave the average values. Sample crystallinity was determined by integrating the enthalpy peak from 30 to 230 °C and normalizing it with the enthalpy of melting of 100% crystalline polyethylene, 291 J/g according to the following equation:

(4) $$X_{c}\left(\%\right)=\frac{\Delta H_{c}}{\left(1-\emptyset \right)\Delta H_{m}^{o}}\cdot 100$$

where Δ*H_c_* is the apparent enthalpy of sample crystallization, Δ*H°_m_* is the melting enthalpy of 100% crystalline UHMWPE and ∅ is the weight fraction of CNF and paraffin oil in the UHMWPE composites.

## 3. Results

This In order to evaluate the tribological behavior during the ageing period in SSF of nanocomposites and of UH sample, a wear test with a morphological study has been performed.

**Figure 1:**
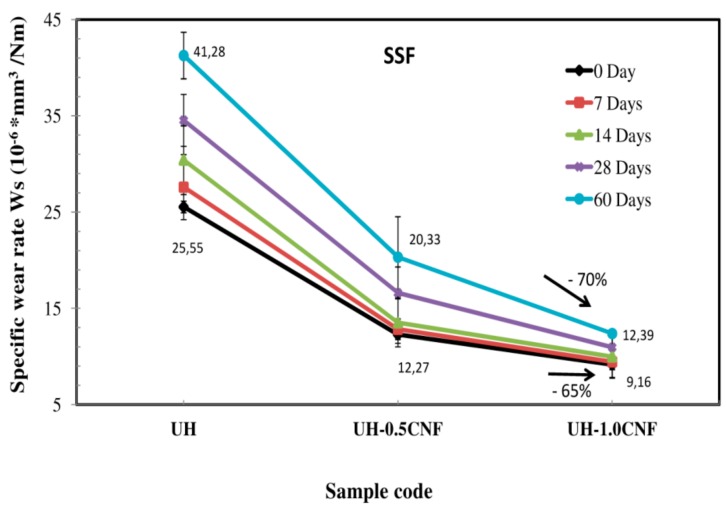
Specific wear rate of UH, UH-0.5CNF, UH-1.0CNF samples for 60 days of ageing in SSF.

Results are presented in Figure 1 which shows that:

1. Nanocomposites exhibited a much higher wear resistance than pure UH: the higher was the CNF load, the higher was the wear rate reduction, as before as after the ageing period of 60 days, due to the lubricant action of CNF [21]. In percentage, UH-1.0CNF nanocomposite exhibits the lowest wear rate at 0 day, not aged (9.16 × 10^−6^ mm^3^/Nm), which is about 65% lower than pure UH (25.55 × 10^−6^ mm^3^/Nm). Similarly, after 60 days of ageing in SSF, the wear reduction in the nanocomposite UH-1.0CNF is about 70% (from 41.28 × 10^−6^ to 12.39 × 10^−6^ mm^3^/Nm).
2. The specific wear rate in SSF (Ws) value of each sample increases during the ageing time; anyway, the increase is smaller with increasing of the CNF amount in the nanocomposite. After 60 days of ageing in SSF, in fact, Ws in pure UH improves of +64% (from 25.55 × 10^−6^ mm^3^/Nm up to 41.28 × 10^−6^ mm^3^/Nm), while in the nanocomposites UH-0.5CNF and UH-1.0CNF it grows of +65% (from 12.27 × 10^−6^ mm^3^/Nm up to 20.33 × 10^−6^ mm^3^/Nm) and of +35% (from 9.16 × 10^−6^ mm^3^/Nm up to 12.39 × 10^−6^ mm^3^/Nm), respectively.

Thus, the UH-1.0CNF sample was selected for the subsequent investigations; UH was used as control sample.

At parity of CNF amount (1 wt.%), the nanocomposites prepared by Panin et al. [22] (without PO) exhibited a wear reduction of about −54% in air that was lower than that of our sample UH-1.0CNF prepared with PO assisted mixing technique (−65%). This suggests that paraffin oil presence is important to better disperse the filler in the polymeric matrix during the nanocomposite processing and to improve the lubricant action of CNF.

**Figure 2:**
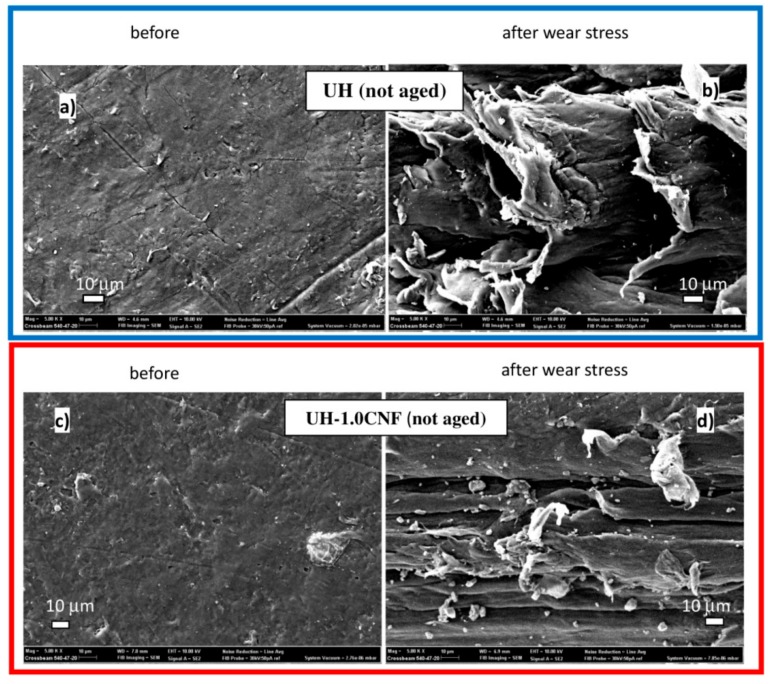
SEM micrographs at 5K× of the not aged external surfaces of: pristine UH before (**a**) and after the wear stress (**b**); pristine UH-1.0CNF before (**c**) and after the wear stress (**d**).

SEM observation (at 5K×) analyzed the morphological changes induced by the wear action on the studied materials before ageing (Figure 2). The surface of UH-1.0CNF nanocomposite is smoother and with fewer defects than pure UH, which consists of more scratches and pitting (Figure 2a,c). This is typical of an abrasive wear because the polymeric surface is invested by the stone pin of higher hardness. Cracks start from the surface irregularities and then they propagate on the surface for the repeated deformation of the applied load. Small particles are produced and then joining together into chips by the heat generated as a result of friction and sliding between the sample and the stone pin. Both surfaces of UH and of UH-1.0CNF appear damaged with discontinuous broken paths and with very fine particles, few microns wide, randomly placed between one path and another; anyway the damage is visibly much higher in the pure UH than in the nanocomposite (Figure 2b,d). These results are in accordance with the literature data which states that polyethylene typically suffers fatigue damage (pitting and delamination), adhesive and abrasive wear. Adhesive and abrasive wear are the main processes responsible for most of wear debris, which then cause osteolysis and loosening [4,32].

**Figure 3:**
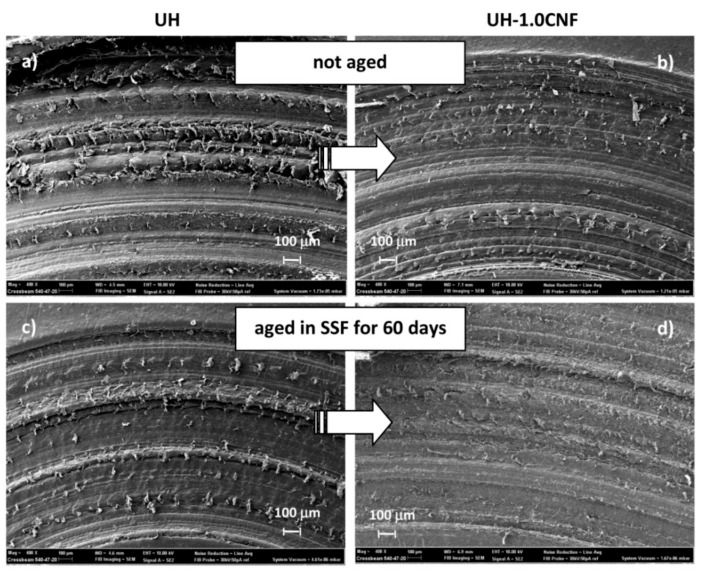
SEM micrographs at 400× of wear tack of UH (**left**) and UH-1.0 CNF (**right**) before (**a**), and after 60 (**b**) days of immersion in SSF.

During the ageing process, the surface of pure UH became softer as a result of SSF penetration; this increases the wear rate for the abrasive action of the harder stone pin with increasing the ageing time. This is visible in SEM micrographs of Figure 3a,c, which show the morphological changes of the entire wear track in pure UH and UH-1.0 CNF nanocomposite (not aged). Cords of debris, separated from each other, are formed inside the wear path. Pure UH shows a wear track in which the external edges are wrinkled with large areas of debris that are the result of the debris paths collection. Wear track in the UH-1.0CNF is visibly less pronounced than those in pure UH, before as well as after the ageing in SSF for 60 days (Figure 3b,d). The typical adhesive and abrasive wear is, in fact, highly reduced in the nanocomposite with respect to pure UH. SSF penetration is instead hidden by CNF, which close the porosity of UHMWPE and prevent SSF to penetrate inside the polymeric matrix (Figure 3b,d) [33]. This result suggests that in both cases the nanocomposite is higher wear resistant than UH, especially after SSF ageing. These observations are in agreement with wear results presented in Figure 1.

**Figure 4:**
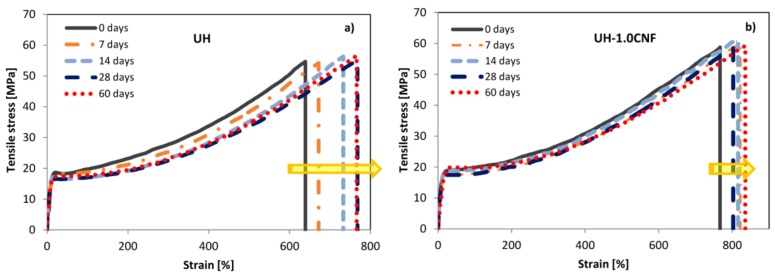
Representative stress/strain curves of UH (**a**) and UH-1.0CNF (**b**) during the 60 days of immersion time.

Tensile test results of Figure 4 confirm that SSF diffuse inside the polymeric bulk, softening the material. In fact, UH samples change only the elongation at break during the 60 days, from 630% to 780% (+24%), (Figure 4a). The curves of the UH-1.0CNF samples (Figure 4b) do not change so much in strain during ageing period (from 794% to 836%, growing of +5%), showing enhanced sample’s homogeneity and stability, compared to pure UH.

**Figure 5:**
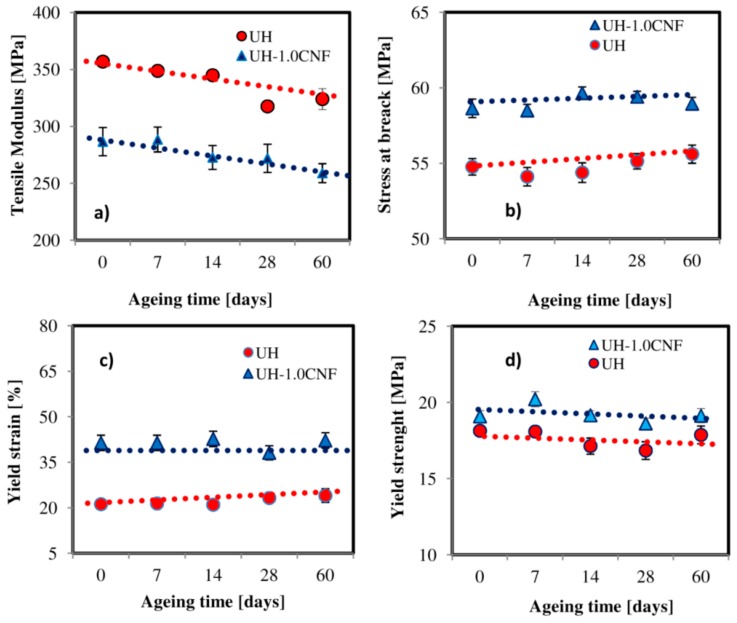
Tensile Modulus (**a**), Stress at break (**b**), Yield strain (**c**) and Yield strength (**a**) and strain at break (**b**) of UH and UH-1.0CNF samples during the 60 days of ageing time.

Furthermore, we can see the parallel trend of the mechanical parameters during all the ageing period (Figure 5). This suggests that SSF action occurs in a similar way in the two materials, involving mainly the polyethylene matrix. Paraffin oil decreases the stiffness of pure UHMWPE (from 357 MPa to 272 MPa), increasing its ductility [25]. It also facilitates the homogeneous dispersion of the filler inside it, otherwise difficult by the high viscosity of UHMWPE. Therefore, the presence of the filler (CNF, 1% by weight) re-increases the stiffness of the nanocomposite, as typically one can expect from a carbon nanofiller (from 272 MPa to 287 MPa)*.* So, nanocomposite tensile modulus before ageing (287 MPa) is lower than that one of pure UH (357 MPa), Figure 5a. Both modulus values then decrease with increasing the ageing time: after 60 days they became 236 MPa and 324 MPa, in nanocomposite and pure UH, respectively. Yield strength, yield strain, stress at break of UH and UH-1.0CNF are about 19 MPa, 41% and 59 MPa, respectively before immersion; these parameters are always higher than in pure UH (about 18 MPa, 22% and 55 MPa) (see Figure 5b–d).

**Figure 6:**
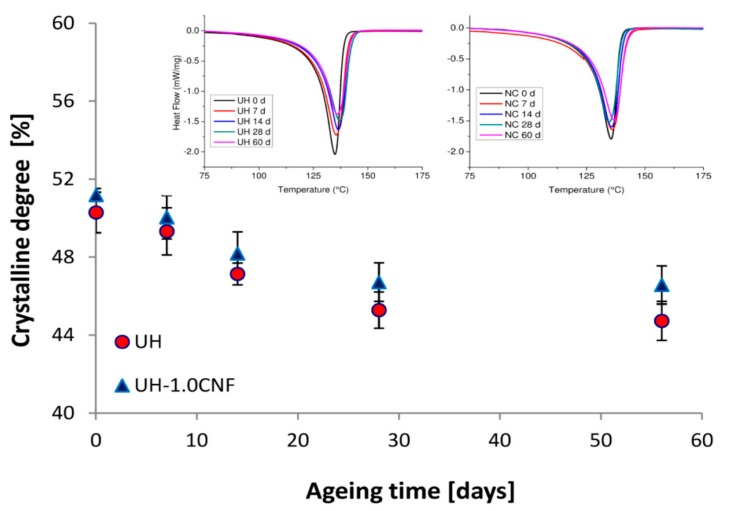
Crystalline degree in SSF of UH and UH-1.0CNF samples during the 60 days of ageing time with the DSC peaks in the insert.

The not aged nanocomposite exhibited a different organization in terms of crystalline degree (51.2%) with respect to pure UH (50.3%) [22]. This because CNF acts as heterogeneous nucleating agent to facilitate the re-crystallization of UHMWPE and improving its structural order [34]. Crystalline order of nanocomposite remains always just a little higher than pure UH during all the ageing time (Figure 6). Both samples decrease in a similar way: SSF starts to diffuse inside the polymeric bulk, increasing the disorder. The values progressively decrease to a value of 46.5% (nanocomposite) and of 44.7% (pure UH) at 60 days of immersion time. S. Ge et al. [10] observed that SSF penetrate into UHMWPE structure inducing a swelling and weight gain. As a consequence, internal disorder increases with a weakening and destruction of macromolecular secondary links between cohesion chains of polyethylene, decreasing its overall mechanical cohesion. In accordance with these Authors, we have observed an improvement in weight (hence in density, see Table 2) and a decrease in the stiffness of the material’s bulk. This confirms that SSF molecules diffuse in polymeric matrix; consequently, stiffness decreases while deformability improves. Ageing effect in the nanocomposite is similar than that in pure UH sample because all the changes occur in the polymeric matrix. This is easily visible by the parallel behaviour.

It must be taken into account that ageing effects are highly accelerated by mechanical stress action during the joint action. Environmental Stress Cracking (ESC) is a common cause of failure in plastic materials during their use [35]; it is due to the action of both stress and surface-active substances, known as “stress-cracking agents”, such as lubricants, which accelerate the process of macroscopic brittle-crack formation [36]. Generally, ESC involves stress enhanced absorption, permeation, local yielding, cavitations, fibrillation and fracture. Under stress, yielded areas cavities and fibrillate areas, become crazes. In high molecular weight polymers (such UHMWPE) formed crazes can progress to cracks; in lower molecular weight polymers, instead, crack formation occurs directly, without any craze formation [37]. Semi-crystalline polymers, like UHMWPE, are more resistant to the fluid diffusion, than amorphous ones. The fluid absorption softens the polymer and reduces yield strength. In this paper we have not observed any appreciable yield strength change, suggesting the necessity of longer term ageing tests.

All the above-discussed results highlighted as both PO and CNFs are important for a better wear resistance of our nanocomposites based on biomedical grade UHMWPE. About their biocompatibility or cytotoxicity, PO is a mineral oil used in cosmetics and for other medical purposes. So, PO has a good compatibility with all the human body [38]. Instead, carbon nanofiller could be dangerous, since it could migrate in the human body. Graphite is the most dangerous for its dangling carbon bonds (highly reactive) and for possible traces of residual catalysts. Fullerenes and single wall carbon nano-tubes and are instead considered safer. Anyway, until now, a detailed and reasonable study about this aspect is still not known [39].

In wear application of nanocomposites anyway, eventual migration of the filler is strictly related to the wear features of the system. The more resistant to wear the material is, the less is the potential amount of carbon filler which can go in the biological environment. Then, a great improvement of wear resistance on implants can surely reduce the danger about adverse effect on human body. Furthermore, the next step on this research will be devoted on using different fillers, like graphene or graphene oxide, which for their chemical and physical nature, is considered highly promising for which concerns both performance in wear resistance and biocompatibility for the studied applications [40,41]. The above-discussed results suggest that several other aspects of this research need to be studied: so, further investigations will be devoted to the:

- evaluation of tribological behavior of nanocomposites with higher CNF loads;
- evaluation of SSF action during a long-term wear stress;
- study of the filler stability inside the polymeric matrix and their release;
- study of the nanocomposite degradation process in SSF and its consequent failure modes.

Finally, considering that proteins are absent in SSF, a future study on the best nanocomposite formulation will be repeated in bovine serum (natural lubricant). By this way, we can distinguish the effect of HA (present in SSF) by that of the proteins upon the UHMWPE. In fact, as discussed in the introduction section, it is already known that UHMWPE is highly affected by protein layer since the biomolecules are adsorbed and diffuse within it and proteins/lipids role in wear mechanism of UHMWPE is still not completely understood [42].

## 4. Conclusions

In this paper we analysed the tribological behaviour by means of a wear test of nanocomposites based on biomedical grade UHMWPE, paraffin oil, and carbon nanofiber before and during an ageing in SSF for 60 days at 37 °C in order to reproduce the organic environment of an artificial joint. Results were compared to pure UH, the reference sample. Mechanical and physical characterization tests have been performed on the nanocomposite with the aim to explain the reasons of the different nanocomposites degradation resistance in SSF compared to pure UH sample, used as reference. Experimental results suggested that:

- ageing resistance of the nanocomposites is higher than pure UH and it improves with the CNF load;
- SSF penetrates into the structure of UHMWPE inducing a weight gain with decreasing its overall mechanical cohesion, crystalline order and stiffness;
- both PO and CNF improves nanocomposite’s homogeneity compared to pure UH: they close the porosity of UHMWPE, preventing SSF to penetrate inside the polymeric matrix;
- the UH-1.0CNF sample exhibits a higher wear resistance in SSF of about 65% (before ageing) and of about 70% (after 60 days of ageing), with respect to pure UH.
- Wear resistance in dry (before ageing) was higher than that obtained without PO (in comparison also with literature data). Hence PO assisted mixing is useful for nanocomposite preparation.

The above-discussed encouraging results demonstrated that the employment of such formulation of nanocomposite material gives improved results in terms of material stability, especially in SSF, compared to pure UH.

## Figures and Tables

**Table 1:** Composition of simulated synovial fluid, Simulated Synovial Fluid (SSF) (**left**) and chemical formula of Hyaluronic acid (HA) (**right**).

**Inorganic Electrolyte**	**Concentration (mM)**	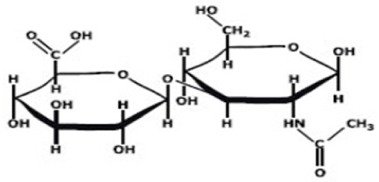
Na^+^	153.1	
K^+^	4.2	
Cl^−^	139.6	
Phosphate buffer	9.6	

**Table 2:** Density values of pure UH and of the nanocomposites at different immersion times.

Ageing Time (Days)	Density (g/mL)		
	UH	UH-0.5CNF	UH-1.0CNF
0	0.866 ± 0.002	0.844 ± 0.008	0.862 ± 0.004
7	0.866 ± 0.004	0.849 ± 0.004	0.865 ± 0.003
14	0.871 ± 0.003	0.856 ± 0.009	0.869 ± 0.007
28	0.877 ± 0.008	0.861 ± 0.004	0.870 ± 0.009
60	0.878 ± 0.006	0.867 ± 0.004	0.873 ± 0.005

## References

[1] Kurtz S.M. (2009). UHMWPE Biomaterials Handbook.

[2] Di Fiore N. (2016). Figuring out Why Artificial Joints Fail-Rush Study Aims to Extend Implant Longevity.

[3] Yousef S., Berber M. (2016). Polymer Nanocomposite Artificial Joints. Carbon Nanotubes–Current Progress of Their Polymer Composites.

[4] Baena J.C., Wu J., Peng Z. (2015). Wear performance of UHMWPE and reinforced UHMWPE composites in arthroplasty applications: A review. Lubricants.

[5] Pokorny D., Slouf M., Vesely F., Fulin P., Jahoda D., Sosna A. (2010). Distribution of UHMWPE wear particles in periprosthetic tissues of total hip replacements. Acta Chir. Orthop. Tr..

[6] Yoshinori S., Teruo M., Jian C. (1998). Effect of synovia constituents on friction and wear of ultra-high molecular weight polyethylene sliding against prosthetic joint materials. Wear.

[7] Xue Y., Wu W., Jacobs O., Schädel B. (2006). Tribological behaviour of UHMWPE/HDPE blends reinforced with multi-wall carbon nanotubes. Polym. Test..

[8] Saikko V., Shen M. (2010). Wear comparison between a dual mobility total hip prosthesis and a typical modular design using a hip joint simulator. Wear.

[9] Smith S.L., Li B.L., Buniya A., HoLin S., Scholes S.C., Johnson G., Joyce T.J. (2015). In vitro wear testing of a contemporary design of reverse shoulder prosthesis. J. Biomech..

[10] Ge S., Kang X., Zhao Y. (2011). One-year biodegradation study of UHMWPE as artificial joint materials: Variation of chemical structure and effect on friction and wear behavior. Wear.

[11] Takadama H., Mizuno M. (2006). A simulated synovial fluid for wear characterization of artificial hip joints by a hip joint simulator. Key Eng. Mater..

[12] Choudhury N.R., Kannan A.G., Dutta N.K., Friedrich K., Schlarb A.K. (2008). Novel nanocomposites and hybrids for high-temperature lubricating coating applications. Tribology of Polymeric Nanocomposites-Friction and Wear of Bulk Materials and Coatings.

[13] Galetz M.C., Bla T., Ruckdäschel H., Sandler J.K.W., Altstädt V., Glatzel U. (2007). Carbon nanofibre-reinforced ultrahigh molecular weight polyethylene for tribological applications. J. Appl. Polym. Sci..

[14] Chen J., Cao Y., Li H. (2006). An investigation on wear mechanism of POM/LLDPE blends. J. Appl. Polym. Sci..

[15] Roba M., Naka M., Gautier E., Spencer N.D., Crockett R. (2009). The adsorption and lubrication behavior of synovial fluid proteins and glycoproteins on the bearing-surface materials of hip replacements. Biomaterials.

[16] Balazs E.A., Watson D., Duff I.F., Roseman S. (1967). Hyaluronic acid in synovial fluid. I. Molecular parameters of hyaluronic acid in normal and arthritis human fluids. Arthritis Rheumatol..

[17] Gispert M.P., Serro A.P., Colaço R., Saramago B. (2006). Friction and wear mechanisms in hip prosthesis: Comparison of joint materials behaviour in several lubricants. Wear.

[18] Yan Y., Neville A., Dowson D. (2007). Biotribocorrosion of CoCrMo orthopaedic implant materials—Assessing the formation and effect of the biofilm. Tribol. Int..

[19] Visco A.M., Campo N., Torrisi L., Cristani M., Trombetta D., Saija A. (2008). Electron beam irradiated UHMWPE: Degrading action of air and hyaluronic acid. Bio-Med. Mater. Eng..

[20] Zhang M., Pare P., King R., James S.P. (2007). A novel ultra high molecular weight poly-ethylene—Hyaluronan microcomposite for use in total joint replacements.II. Mechanical and tribological property evaluation. J. Biomed. Mater. Res. Part A.

[21] Azam A.M., Ali A., Khan H., Yasin T., Mehmood M.S., Sara Qaisar A., Khan N., Mukhtar E.A. (2016). Analysis of degradation in UHMWPE a comparative study among the various commercial and laboratory grades UHMWPE. Proceedings of the 14th International Symposium on Advanced Materials (ISAM 2015), National Centre for Physics, Islamabad, Pakistan, 12–16 October 2015.

[22] Panin S.V., Kornienko L.A., Nguyen D.A., Alexenko V.O., Ivanova L.R., Eduard S., Gorkunov V.E., Sunder R. (2017). Enhancement of mechanical and tribotechnical properties of polymer composites with thermoplastic UHMWPE and PEEK matrices by loading carbon nanofibers/nanotubes. Proceedings of the 11th International Conference on Mechanics, Resource and Diagnostics of Materials and Structures, AIP Conference Proceedings Mechanics, Resource and Diagnostics of Materials and Structures (Mrdms-2017).

[23] Kurtz S.M. (2004). The UHMWPE Handbook: Ultra-High Molecular Weight Polyethylene in Total Joint Replacement.

[24] Affatato S. (2012). Wear of Orthopaedic Implants and Artificial Joints.

[25] Visco A., Yousef S., Galtieri G., Nocita D., Njuguna J. (2016). Thermal, Mechanical and Rheological behaviors of nanocomposites based on UHMWPE/Paraffin Oil/Carbon Nano filler obtained by using different dispersion techniques. JOM-Springer.

[26] Liu S.L., Chen J.Y., Cao Y. (2015). Effect of solid paraffin on the integrity of welded interfaces and properties of ultra-high molecular weight polyethylene. Polym. Sci. Ser. A.

[27] Wood W.J., Maguire R.G., Zhong W.H. (2011). Improved wear and mechanical properties of UHMWPE–carbon nanofiber composites through an optimized paraffin-assisted melt-mixing process. Compos. Part B.

[28] Puertolas J.A., Kurtz S.M. (2014). Evaluation of carbon nanotubes and graphene as reinforcements for UHMWPE-based composites in arthroplastic applications: A review. J. Mech. Behav. Biomed. Mater..

[29] Yousef S., Visco A., Galtieri G., Nocita D., Espro C. (2017). Wear behaviour of UHMWPE reinforced by carbon nanofiller and paraffin oil for joint replacement. Mater. Sci. Eng. C.

[30] Marques M.R.C., Loebenberg R., Almukainzi M. (2011). Simulated Biological Fluids with Possible Application in Dissolution Testing. Dissolut. Technol..

[31] Agarwal G., Patnaik A., Sharma R.K. (2013). Parametric optimization and three-body abrasive wear behavior of sic chopped glass fiber reinforced epoxy composites. Int. J. Compos. Mater..

[32] Chakrabarty G., Vashishtha M., Leeder D. (2015). Polyethylene in knee arthroplasty: A review. J. Clin. Orthop. Trauma.

[33] Golchin A., Wikner A., Emami N. (2016). An investigation into tribological behaviour of multiwalled carbon nanotube/graphene oxide reinforced UHMWPE in water lubricated contacts. Tribol. Int..

[34] Chiu H.T., Wang J.H. (1998). Characterization of the rheological behavior of UHMWPE gels using parallel plate rheometry. J. Appl. Polym. Sci.

[35] Hough M.C., Wright D.C. (1996). Two new test methods for assessing environmental stress cracking of amorphous thermoplastics. Polym. Test..

[36] Lagaron J., Pastor J., Kip B. (1999). Role of an active environment of use in an environmental stress crack resistance (ESCR) test in stretched polyethylene: A vibrational spectroscopy and a SEM study. Polymer.

[37] Wright D. (1996). Environmental Stress Cracking of Plastics.

[38] Bin Y., Yamanaka A., Chen Q., Xi Y., Jiang X., Matsuo M. (2007). Morphological, electrical and mechanical properties of ultrahigh molecular weight polyethylene and multi-wall carbon nanotube composites prepared in decalin and paraffin. Polym. J..

[39] Fiorito S., Serafino A., Andreola F., Togna A., Togna G. (2006). Toxicity and biocompatibilità of carbon nanoparticles. J. Nanosci. Nanotech..

[40] Chen Y., Qi Y., Tai Z., Yan X., Zhu F., Xue Q. (2012). Preparation, mechanical properties and biocompatibility of graphene oxide/ultrahigh molecular weight polyethylene composites. Eur. Polym. J..

[41] Aliofkhazraei M., Ali N., Milne W.I., Ozkan C.S., Mitura S., Gervasoni J.L. (2016). Graphene. Science Handbook: Applications and Industrialization.

[42] Fröhlich S.M., Dorrer V., Archodoulaki V.-M., Allmaier G., Marchetti-Deschmann M. (2014). Synovial fluid protein adsorption on polymer-based artificial hip joint material investigated by MALDI-TOF mass spectrometry imaging. EuPa Open Proteomics.

